# Hospital Meetings, &c.

**Published:** 1900-07-28

**Authors:** 


### HOSPITAL MEETINGS, &C.

### DENTAL HOSPITAL OF LONDON.

On the 18th inst. Mr. F. A. Bevan distributed the prizes gained during the past year by the students of the school department of the Dental Hospital of London. The ceremony took place at a conversazione, held in the Royal Institute Galleries, Prince's Hall, Piccadilly. Mr. Morton Smale, the Dean, presented a satisfactory report of the year's work, and said that both the work of the appliance department and that of teaching mechanical dentistry to students continued to go on successfully. He urged that all interested in the welfare of the dentist should use their influence with members of Parliament to vote for the retention of the clauses in the Companies Bill which prohibit the formation of companies for the practice of dentistry and other branches of surgery. Dr. Walker said the new hospital building in Leicester Square was far advanced towards completion, and the committee hoped to begin work in it in October. Mr. Bevan, having distributed the awards, spoke of the usefulness of the work which the hospital performed, and said there were few institutions in which he took such a deep interest. At the new building greatly improved facilities would be provided, and he trusted that increased support would be forthcoming from the public for its maintenance.

### QUEEN CHARLOTTE'S HOSPITAL.

The half-yearly meeting of Governors of Queen Charlotte's Hospital was held last week, by the kindness of Lord Portman, at 22, Portman Square. Lord Portman himself was in the chair.

The Earl of Hardwicke, chairman of committee, gave a brief statement of the present financial condition of the charity. He wished to state the view of the committee of which he was chairman. With regard to the Midwives Bill, they welcomed it, believing it to be urgent; and they hoped that, when it passed into law, the certificate given by the hospital should be a guarantee for the registration of midwives trained there.

H.R.H. the Duke of Cambridge moved the following resolution:—

"That this meeting desires to express its warm approval of the success which has attended the efforts made by the committee to provide increased and improved accommodation for the growing number of patients seeking the aid of the charity, and cordially endorses the appeal for additional support to enable the committee to carry out the further work necessary for the complete renovation of the hospital."

His Royal Highness said that this hospital rendered a great deal of benefit to the services, both naval and military. This could at once be shown by reference to the number of soldiers and reservists' wives received this year; the total number was 92. Nowadays old institutions were left very much in the cold; new ones were kept going by subscriptions, and it sometimes happened that the new ones were less worthy of support than the old. Anything that was good for the public service should be supported by the public. The demand on the public was great just now, and he did not see that it would be diminished for some time yet. This was a charity in which his family had always taken a deep interest. Queen Charlotte had been patroness, and his father, the late Duke of Cambridge, had been president. Further improvements would be made in the hospital if the necessary means were forthcoming.

The resolution was seconded by Lord Hugh Cecil, who dwelt upon the importance of the medical school in connection with the hospital. In medical and surgical science there had been a degree of progress quite unparalleled. The growth of medical science depended on the efficiency of medical schools, which formed a very important part of the educational machinery of the country, and it might be hoped that medical science would go on with not only equal, but accelerated pace. To make this possible, however, it was necessary that funds should be forthcoming.

The resolution was carried unanimously.

### EAST LONDON HOSPITAL FOR CHILDREN.

Lord Falkland presided at the half-yearly Court of Governors of the East London Hospital for Children and Dispensary for Women at Shadwell on July 25th. The report presented by the Board of Management gave the receipts for the six months ended June 30th as £5,535, as compared with £5,654 in the corresponding half of 1899. Annual subscriptions showed an increase of £78, and donations an increase of £747, but legacies fell off by close on £1,000. The festival dinner in May produced contributions of some £1,400, including £75 collected by the Ladies' Association. The proceeds of a reading by Sir Squire Bancroft at St. Martin's Town Hall in January last were £250. £100 of this was allotted to the Sea-side Branch at Bognor; but even with this the contributions towards that home amounted only to £269, which the board pointed out was not satisfactory, since an expenditure for the six months of over £400 had been incurred for maintenance. The deficiency was the more to be regretted since the year's expenditure will be materially augmented by the increase in the cost of most articles of consumption, particularly coal and gas. The new ward of four beds for surgical cases was now being furnished, and would be ready for occupation immediately. The board would have preferred to wait until a larger portion of the estimated additional £300 a year in expenses which this would entail had been provided by new annual subscriptions, but the pressure on the beds had decided them to open it at once. An appeal was made for contributions toward the maintenance of the donkey and cart for the use of the small patients, the £20 given by Mr. E. Schenk in 1899 having been exhausted. The purchase of the plot of land adjoining the hospital having been completed, plans would now be matured for the erection of the new laundry, mortuary, &c., so urgently needed. The provision of additional domestic, nursing, and other accommodation was also under consideration. The new operating theatre had been opened during the half-year and was giving satisfaction to the surgeons. On the medical staff Mr. A. Herbert Hayes had succeeded Mr. C. Frank Steele as medical officer for the casualty department, being himself succeeded as house surgeon by Mr. Sydney Vere Pearson. The number of in-patients treated during the six months was 739, as compared with 712 in the corresponding half-year. The new out-patients numbered 17,319 as against 16,471, with attendances of 38,422 against 38,224 in 1899. In the convalescent home there were on January 1st 21 patients, and 158 had been sent down during the six months. The Chairman briefly moved the adoption of the report, which was carried, and the proceedings terminated.

